# Identification of a novel nonsense mutation and a missense substitution in the *AGPAT2* gene causing congenital generalized lipodystrophy type 1

**DOI:** 10.1016/j.ejmg.2012.07.011

**Published:** 2012-11

**Authors:** Amirreza Haghighi, Maryam Razzaghy-Azar, Ali Talea, Mahnaz Sadeghian, Sian Ellard, Alireza Haghighi

**Affiliations:** a The Hospital for Sick Children, University of Toronto, Toronto, Ontario, Canada; b Endocrinology and Metabolism Research Center, Tehran University of Medical Sciences, Tehran, Iran; c Pediatric Gastroenterology Department, H. Aliasghar Hospital, Tehran University of Medical Sciences, Tehran, Iran; d Institute of Biomedical and Clinical Science, Peninsula College of Medicine and Dentistry, University of Exeter, Exeter, UK; e Wellcome Trust Centre for Human Genetics, University of Oxford, Roosevelt Drive, Headington, Oxford OX3 7PS, UK

**Keywords:** Congenital generalized lipodystrophy, CGL, Berardinelli-Seip syndrome, AGPAT2

## Abstract

Congenital generalized lipodystrophy (CGL) is an autosomal recessive disease characterized by the generalized scant of adipose tissue. CGL type 1 is caused by mutations in gene encoding 1-acylglycerol-3-phosphate *O*-acyltransferase-2 (*AGPAT2*). A clinical and molecular genetic investigation was performed in affected and unaffected members of two families with CGL type 1. The *AGPAT2* coding region was sequenced in index cases of the two families. The presence of the identified mutations in relevant parents was tested. We identified a novel nonsense mutation (c.685G>T, p.Glu229*) and a missense substitution (c.514G>A, p.Glu172Lys). The unaffected parents in both families were heterozygous carrier of the relevant mutation. The results expand genotype–phenotype spectrum in CGL1 and will have applications in prenatal and early diagnosis of the disease. This is the first report of Persian families identified with *AGPAT2* mutations.

## Highlights

► First diagnosis of congenital generalized lipodystrophy type 1 in Persian population. ► Molecular analysis identified a novel nonsense mutation and a missense substitution in the *AGPAT2*. ► The patients did not have diabetes mellitus or hyperinsulinemia. ► The mutations found are candidates for CGL screening. ► The results expand the knowledge about the genotype–phenotype correlations in CGL.

## 1 Introduction

Congenital generalized lipodystrophy (CGL) is a rare metabolic disease, transmitted in an autosomal recessive mode. The worldwide prevalence of CGL has been estimated at one in 10 million [1]. Interestingly, Rajab et al. reported a significantly higher incidence of one in 25,000 births in Oman [2].

The disease is characterized by a near total loss of adipose tissue throughout the body from birth, resulting in a marked and generalized muscular appearance [3]. The other common clinical features include muscular hypertrophy, resistance to insulin, hypertriglyceridemia, acanthosis nigricans, hyperandrogenism, hepatosplenomegaly and acromegaly [4,5]. Studies indicated that CGL increases the risk of development of type 2 diabetes mellitus and cardiovascular diseases [6,7]. Mortality in CGL is mainly due to liver, kidney and heart impairment [8]. Four genes have been identified to cause different types of CGL; *AGPAT2* (9q34) [9], *BSCL2* (11q13) [10], *CAV1* (7q31.1) [11] and *PTRF* (17q21.2) [12]. Two major forms of congenital generalized lipodystrophy, CGL1 (caused by AGPAT2 mutations) and CGL2 (due to mutation in *BSCL2*), account for 95% of all cases [13]. They have some similar and some distinct clinical features. For example, lack of metabolically active adipose tissue is a common finding in both forms but CGL2 patients lack mechanical adipose tissue as well [14]. Risk of some conditions such as cardiomyopathy and mental retardation is increased in patients affected by CGL2 [3,15].

*AGPAT2* encodes an enzyme 1-acyl-glycerol-3-phosphate acyltransferase-β, which has a role in the synthesis of triglycerides by catalyzing the conversion of 1-acylglycerol-3-phosphate (lysophosphatidic acid [16]) to 1,2-diacylglycerol-3-phosphate (phosphatidic acid) [17, 18]. *AGPAT2* is highly expressed in the adipose tissue. Mutations that cause deficiency of *AGPAT2* protein result in a decrease of triglyceride or phospholipid biosynthesis that can lead to lipodystrophy [19].

In this study, we investigated clinical features of the first families with congenital generalized lipodystrophy in Persian population and identified the disease causing *AGPAT2* mutations in these two unrelated cases.

## 2 Materials and methods

### 2.1 Patients and families

Two Persian families with congenital generalized lipodystrophy were studied. Written consent for participation in clinical and molecular investigation was obtained from all members of both families. All family members underwent a comprehensive physical examination.

### 2.2 Molecular analysis

Peripheral blood samples were collected from all participating family members. Genomic DNA was extracted using Qiagen mini kit (Qiagen, Hilden, Germany) according to manufacturer's instructions.

Direct sequencing of *AGPAT2* gene was performed using the DNA samples of patients from both families. Six exons and flanking sequence of *AGPAT2* gene were amplified by PCR using gene-specific primers described previously [9]. The PCR products were directly sequenced using standard methods on a Big Dye Terminator Cycler Sequencing Kit v3.1 (Applied Biosystems, Warrington, UK). The reactions were analyzed on an ABI 3730 (Applied Biosystems). Sequences were compared with the published reference sequence (NM_006412.3) using Mutation Surveyor v3.2 (SoftGenetics, State College, PA). The unaffected parents were tested for the presence of mutations identified in the probands.

## 3 Results

### 3.1 Clinical description

*Family 1*: The male proband (case1) was admitted to the pediatric surgery department at the age of 2 months because of a mass in his right groin. He was diagnosed with right inguinal hernia and operated. He was then referred to the department of pediatric gastroenterology because of the abdominal distention and hepatosplenomegaly.

The patient was born at term after an uneventful pregnancy and delivery. His weight, height and head circumference at birth were 3200 g, 50 cm and 34 cm, respectively. He was the only child of nonconsanguineous parents. No similar condition was reported in the family.

**Fig. 1:**
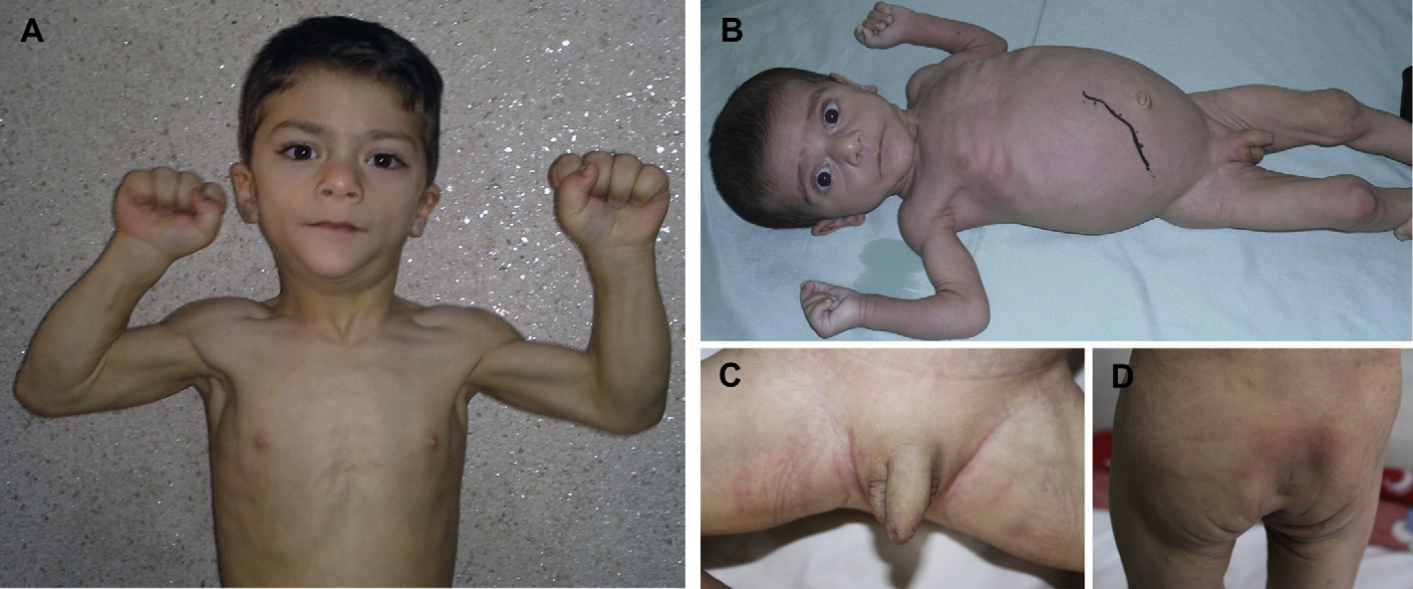
Clinical features of the patients. A: *Case1*. Generalized lack of subcutaneous fat, marked muscular hypertrophy, hollow cheeks, and broad hands. B–D: *Case2*. Complete lipodystrophy (B and D), muscular hypertrophy (B), protuberant abdomen and hepatosplenomegaly (black mark for liver border) (B), enlarged hands (B) unusual facial features (B) and crassitude of the penis (B&C).

On physical examination, his clinical features included generalized lack of subcutaneous fat, muscular hypertrophy of all limbs, hollow cheeks, low anterior hair line, prominent orbital ridges and large ears (Fig. 1.A), broad hands and feet with prominent superficial veins and hepatosplenomegaly were noted (Table 1).

Weight, height and head circumference at the age of two months were 5 kg (25–50 centile), 53 cm (25–50 centile), 39 cm (50–75 centile), respectively.

Fasting blood sugar (FBS), blood urea nitrogen (BUN), creatinine (Cr) and electrolytes as well as complete blood count were within normal limits. The results of serum lipids were triglyceride 3000 mg/dL (normal levels for age and sex, 29–99), cholesterol 550 mg/dL (114–203), high-density lipoprotein (HDL) 28 mg/dL and low-density lipoprotein (LDL) 78 mg/dL. Liver function and thyroid function tests were normal.

On imaging, the Z-score of bone mineral density of hip and lumbar spine was normal. Abdominal ultrasonography revealed enlargement of the liver (span, 80 mm) and spleen (55 mm). A 3 mm-stone was seen in the lower pole of the left kidney. Random urine examination for determining the reason of stone revealed the following results: creatinine 30.5 mg/dL, calcium 6 mg/dL, Ca/Cr 0.19 (normal <0.2), uric acid 46 mg/dL, uric acid/Cr 1.5 (normal <1), oxalate 0.26 mg/dL, oxalate/Cr 0.008 (normal <0.02). Cyanide nitroprusside test for cystine was negative.

Liver biopsy was performed and revealed marked fatty infiltration consistent with the diagnosis of congenital generalized lipodystrophy (Fig. 1.B–D).

*Family 2*: The proband (case2) was a 3 months male infant referred with vomiting, abdominal distention and hepatomegaly. The parents were first cousin and the patient was the only child of them. He was born after an uneventful and full-term pregnancy and the birth weight was 3450 g. There was no history of similar condition in the family.

On physical examination, he had an unusual face and generalized lipoatrophy with absence of fat tissue in the face and extremities (Fig. 1.B–D). An apparent hypertrophy was particularly present in calf and thigh muscles. The other dysmorphic features included low anterior hair line, hollow cheeks, prominent orbital ridges, large ears, prominent veins over large hands and feet and protuberant abdomen (Table 1). There was no acanthosis nigricans. The patient had a macropenis with 4 cm in length. The growth parameters at the age of three months, including height, weight and head circumference, were 55 cm (50–75 centile), 5 kg (10–25 centile) and 35 cm (50–75 centile), respectively. Ultrasound sonography demonstrated hepatomegaly of 11 cm span with increased echogenicity. Spleen, bladder and left kidney were normal whereas a mild hydronephrosis and pelvic dilatation (anteroposterior diameter of 16 mm) were seen in the right kidney suggesting ureteropelvic junction obstruction (UPJO). On laboratory investigation, CBC, liver function tests, BUN, Cr and electrolytes were normal. Interestingly, the patient did not have elevated blood sugar (FBS 70 mg/dL) and insulin (7.7 μIU/mL). The homeostatic model assessment (HOMA-insulin resistance) was calculated from the formula (insulin [μIU/mL] × glucose [mmol/L]/22.5) and was 1.51. Other laboratory tests included serum cholesterol 221 mg/dL (114–201), triglyceride levels (non-fasting vs. fasting) 2885 mg/dL and 1400 mg/dL, respectively (29–99), LDL 79 mg/dL, HDL 30 mg/dL, VLDL 106 mg/dL, uric acid 7.8 mg/dL (2–6), ammonia 0.9 μg/mL (0.17–0.8), lactate 47 mg/dL (4.5–20), pyruvate 2 mg/dL (0.3–0.9), T4 18.1 μg/dL (7.2–14.4), TSH 17.2 mIU/L (1.7–9.1) and serum calcium 11.3 mg/dL (8.8–11). Creatine phosphokinase (CPK), lactate dehydrogenase (LDH), serum phosphorus, alkaline phosphatase, PTH, T3 and 25-hydroxy-vitamin D were within the normal ranges.

A liver biopsy showed steatotic changes and was consistent with a diagnosis of non-alcoholic fatty liver disease (NAFLD).

On the basis of these findings, the diagnosis of lipodystrophy was established and treatment with medium-chain triglyceride (MCT)-enriched infant formula was started to control hypertriglyceridemia.

### 3.2 Genetic investigation

Molecular analysis of *AGPAT2* in the proband from family1 (case1) identified a novel nonsense mutation in exon 6 of the gene. This mutation (c.685G>T, p.Glu229*) results in premature termination of the protein in codon 229. The healthy parents of case1 were heterozygous for this novel mutation.

Sequencing of *AGPAT2* in the proband of the second family (case2) revealed a homozygous missense mutation in exon 4; c.514G>A, p.Glu172Lys. This mutation alters a glutamic acid in codon 172 to a lysine. Mutation analysis was performed for the unaffected parents and they were both carries of one copy of mutation c.514G>A.

## 4 Discussion

We investigated clinical and molecular features of congenital generalized lipodystrophy in two new families from Persian population. The clinical diagnosis was made on the basis of characteristic features of total absence of adipose tissue, apparent muscle hypertrophy and hyperlipidemia (Table 1).

**Fig. 2:**
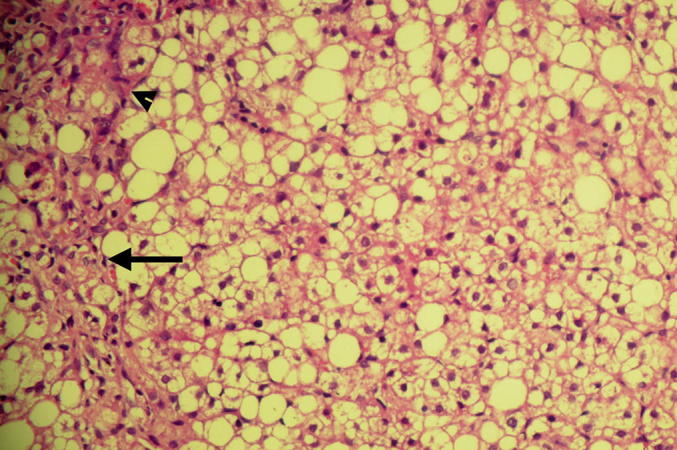
Histopathology of liver biopsy. Severe steatosis of hepatocytes (>66%), infiltration of portal spaces by mononuclear inflammatory cells (arrowhead), evidence of interface hepatitis, fibrous expansion of portal spaces with periportal fibrosis and occasional portal–portal bridging (long arrow). (hematoxylin and eosin).

The histopathological investigation of liver in our patients revealed steatotic changes consistent with a diagnosis of non-alcoholic fatty liver disease (Fig. 2). The histopathologic changes of liver in CGL were investigated previously [20]. The uncharacteristic findings on light microscopy included hepatic steatosis, presence of lipid droplets in hepatocytes which push the cytoplasm to the periphery and sometimes cause nuclear indentation, infiltration of mononuclear inflammatory cells in portal spaces, bile duct proliferation, interface hepatitis, fibrous expansion of portal spaces with occasional portal–portal and portal–central fibrosis, and cirrhosis. In addition, peroxisomes showed catalase activity in catalase staining.

Molecular analysis identified a novel nonsense mutation and a missense substitution in the *AGPAT2* gene responsible for causing congenital generalized lipodystrophy type 1.

In case1, we identified a novel homozygous mutation (c.685G>T) in exon 6 of *AGPAT2* gene that is predicted to cause substitution of a glutamic acid at the position 229 by a nonsense codon, removing 50 amino acids from the end of the protein. Since this mutation is located in the last exon, it is not predicted to result in nonsense mediated decay. AGPAT2 has two highly conserved motifs, NHX4D (amino acids 97–103) and EGTR (amino acids 172–175), shared with other acyltransferases. They have been shown to be critical for the enzymatic activity of the protein [17,21] and nonsense or frame shift mutations affecting one or both these conserved motifs are predicted to be non-functional [22]. Both these motifs are located outside exon 6 and the novel mutation identified by us, c.685G > T, does not affect them. The patient, case1, demonstrated typical CGL phenotypes, suggesting that the C-terminal region of 50 amino acids might also have important role in the protein function. This hypothesis is supported by at least another report of a nonsense mutation from exon 6, c. 676C>T (p.226Gln>X), in a Nepali patient from UK [23], that removes 47 amino acids from the protein. This patient had similar features to case1. In addition, two other mutations, p.L228P and p.252delMRT, were reported in the far carboxyl-end of the protein that retained the critical motifs for acyltransferase activity, but had markedly reduced enzyme activity [9,22].

In case2, the *AGPAT2* mutation c.514G>A in exon 4 caused the missense mutation p.Glu172Lys. The glutamic acid residue is located within the motif EGTR that is conserved through different species [17]. It has been shown that this glutamic acid residue is essential for catalytic function involved in binding of lysophosphatidic acid acyltransferase (LPA) [21]. Magre' et al. [23] reported two families from Turkey and Czech Republic who had children affected by CGL and carried homozygous p.Glu172Lys. Both families had a Turkish ethnic background and based on haplotype analysis, the authors suggested that they might be related [23]. The Turkish patient was from a consanguineous family whereas the parents of the Czech patient were unrelated [23]. Reviewing their clinical details (Seemanova, E and 't Hart, L.M., personal communication in October 2011), most of the findings were overlapping with that of our case, whereas some features were distinct (Table 1). None of our patients had diabetes mellitus and in case2, insulin was measured at the same time with glucose and was within the normal range with no insulin resistance (HOMA-IR, normal), whereas hyperinsulinemia and insulin resistance were observed in both Turkish and Czech patients. The patient from Turkey had normal size of ears and penis, whereas our patients and the Czech patient were born with large ears and genitalia. The distinct findings in the Turkish patient included enlarged heart due to hypertrophic cardiomyopathy, pathological gastroesophageal acid reflux in pH-metry, moderate hypertension and anemia that was responsive to iron therapy. He also had limitation in range of motion of his large joints, particularly of knees and elbows. The Czech patient had hirsutism, periorbital hyperpigmentation, voice hoarseness and acanthosis nigricans in neck, axilla and periumbilical region, of which none was present in two other patients. Hydronephrosis and pelvic dilation were observed only in case2 of the present report.

Treatment with diet and medium-chain triglyceride (MCT)-enriched infant formula was started in both our patients at the time of diagnosis. Changes in the metabolic parameters of the patients during treatment have been summarized in Table 2. On the last follow up of case1, at 3 years of age, the size of his liver and spleen had returned to normal, whereas the other clinical features of CGL were present. None of our patients had diabetes/insulin resistance; however, considering the very young age of our patients, it is important to note that insulin resistance with diabetes can develop later in life.

In conclusion, we have identified two *AGPAT2* mutations in two families with CGL; a novel nonsense mutation (p.Glu229*) and one missense alteration (p.Glu172Lys). The presence of different clinical features in CGL patients emphasizes the phenotypic heterogeneity of this metabolic disorder. This also stresses the importance of genetic testing in CGL that will have both prognostic and genetic counseling applications. Moreover, the results of this study support the idea that besides the two highly conserved motifs, the carboxy terminal part of *AGPAT2* might have an important role in the activity of the protein and its mutation lead to CGL phenotype. This is the first report of CGL cases from Persian population.

Two mutations from *AGPAT2* exon 6 have been previously reported; c. 676C>T (p.226Gln>X, in a Nepali patient from UK) and c.712C>G (p.238Ala>Gly, in a Czech patient) [23].

## Conflict of interest

The authors declare that there is no conflict of interest relevant to this manuscript.

## Figures and Tables

**Table 1:** Clinical features in our patients and previously reported patients with similar mutations.

Clinical features	Case1	Case2	Turkish patient	Czech patient
*AGPAT2* mutation	p.Glu229*	p.Glu172Lys	p.Glu172Lys	p.Glu172Lys
Gender	Male	Male	Male	Male
Generalized lipodystrophy	+	+	+	+
Muscle hypertrophy	+	+	+	+
Hypercholesterolemia	+	+	+	+
Hypertriglyceridemia	+	+	+	+
Diabetes	−	−	+	+
Raised insulin levels	−	−	+	+
Hepatomegaly	+	+	+	+
Splenomegaly	+	−	+	−
Acromegaloid features	+	+	+	+
Large ears	+	+	+	−
Macrogenitalia	+	+	+	−
Inguinal hernia	+	−	−	−
Nephropathy	+	−	−	−
Urine bladder calculus	+	−	−	−
Hydronephrosis	−	+	−	−
Gastroesophageal acid reflux	−	−	+	−
Hypertension	−	−	+	−
Anemia	−	−	+	−
Acanthosis nigricans	−	−	−	+
Hirsutism	−	−	−	+
Voice hoarseness	−	−	−	+
Joint contractures	−	−	−	+
Cardiomyopathy	−	−	−	+
Periorbital hyperpigmentation	−	−	−	+
Mental Retardation	−	−	−	−
Seizures	−	−	−	−

**Table 2:** Changes in the metabolic parameters during treatment.

Patient	Case1				Case2		
Age (months)	2 m	6 m	12 m	36 m	3 m	5 m	8 m
Fasting glucose (mg/dl)	95	90	90	90	80	74	70
Triglycerides (mg/dl)	3000	1600	625	98	2885	90	73
Total cholesterol (mg/dl)	550	280	200	165	221	112	133
